# hsa-miR-340-5p inhibits epithelial–mesenchymal transition in endometriosis by targeting MAP3K2 and inactivating MAPK/ERK signaling

**DOI:** 10.1515/med-2022-0448

**Published:** 2022-03-17

**Authors:** Yiting Wan, Jiami Huang, Yanhua Song, Cancan Gu, Jueying Kong, Ling Zuo, Jing Chen

**Affiliations:** Department of Gynecology, Shanghai Municipal Hospital of Traditional Chinese Medicine, Shanghai University of Traditional Chinese Medicine, Shanghai 200071, China; Department of Gynecology, Shanghai Municipal Hospital of Traditional Chinese Medicine, Shanghai University of Traditional Chinese Medicine, No. 274 Middle Zhijiang Road, Shanghai 200071, China

**Keywords:** endometriosis, hsa-miR-340-5p, MAP3K2, epithelial–mesenchymal transition, MAPK/ERK

## Abstract

Increasing evidence has verified the indispensable effect of microRNAs (miRNAs) in the biological processes of human diseases, including endometriosis. hsa-miR-340-5p was reported to display a low level in patients with endometriosis, but the detailed function of miR-340-5p in endometriosis is unclarified. RT-qPCR was used for the assessment of RNA levels of miR-340-5p and its downstream target genes in endometrial stromal cells (ESCs). Western blotting and Transwell assays revealed that upregulation of miR-340-5p suppressed the migration, invasiveness, and epithelial–mesenchymal transition (EMT) in ESCs. Bioinformatics tools were used to predict miR-340-5p downstream genes. Luciferase reporter assay displayed that miR-340-5p could bind to messenger RNA mitogen-activated protein kinase kinase kinase 2 (MAP3K2). MAP3K2 was targeted by miR-349-5p and could reverse the influence of miR-340-5p. miR-340-5p exerted its impact on the invasive characters of ESCs by inactivating the MAP3K2-mediated MAPK/ERK signaling. In conclusion, miR-340-5p restrains cell migration, invasiveness, and EMT in ESCs by targeting MAP3K2 and inactivating MAPK/ERK signaling.

## 1 Introduction

Endometriosis is a chronic gynecological disorder characterized by the abnormal location of endometrial tissue outside the uterus [1,2]. Endometriosis affects nearly 10% of women of reproductive age, which may result in infertility, pelvic scarring and pain [3,4]. Pharmacotherapy and surgery, mainly laparoscopy, are the typically adopted therapies for endometriosis [5]. Although endometriosis is a benign disease, patients with endometriosis living with pain or the threat of relapse suffer from the great depression and anxiety [6]. Hence, it is significant to figure out the mechanism underlying the progression of endometriosis.

Epithelial–mesenchymal transition (EMT) is a complicated process in which epithelial cells transdifferentiate into mesenchymal cells with migratory and invasive properties [7,8]. EMT is featured with expression decrease in epithelial markers, such as E-cadherin, and expression enhancement in mesenchymal markers, such as N-cadherin [9]. Many studies have elucidated that EMT is involved in the pathogenesis and development of endometriosis [10,11,12]. Alterations in EMT marker proteins have been examined in endometrial stromal cells (ESCs), leading to increased migration and invasiveness and considered as a prerequisite for endometriotic lesion development [13]. Oestrogen treatment can increase N- cadherin expression and decrease E-cadherin expression in endometriosis [14]. MTA1 facilitates the development of endometriosis by inducing EMT via ZEB2 [15].

MicroRNAs (miRNAs) are a group of endogenous RNAs with 18–25 nucleotides [16,17]. Although miRNAs do not encode proteins, they play a significant role in regulating gene expression at the posttranscriptional level [18,19]. The dysregulation of miRNAs has been indicated to implicate in the biological processes of various human diseases, including endometriosis. For example, hsa-miR-199a-3p suppresses the cell motility, contractility and invasiveness in endometriosis [20]. miR-143-3p represses the cell proliferative and invasive abilities in endometriosis by inactivating autophagy [21]. Importantly, miR-340-5p was reported to display a low level in patients with endometriosis [22]. However, the detailed function of miR-340-5p in EMT of ESCs remains unanswered.

Mitogen-activated protein kinase kinase kinase 2 (MAP3K2) encodes the serine/threonine protein kinase family, which preferentially activates other kinases implicated in the mitogen-activated protein kinase (MAPK, originally called ERK) signaling pathway [23]. The MAPK/ERK signaling activation is recognized to play an essential role in the growth and development of endometriotic cells in ectopic sites [24]. In the processes of migration, implantation and invasiveness into the pelvic structures, the aberrant activation of MAPK/ERK signaling leads to the formation of endometriosis and aggravates the condition of patients with endometriosis [25].

This study intended to figure out the detailed function of miR-340-5p in the EMT process, migration and invasion of ESCs. The findings might provide a new perspective for treating endometriosis.

## 2 Materials and methods

### 2.1 Cell culture and transfection

Endometrial stromal cells (ESCs) were purchased from Honsun Biological Technology (Shanghai, China), which were isolated from patients with endometriosis (with no other pathology). ESCs were cultured in Dulbecco’s modified Eagle’s medium (DMEM)/Ham’s F12 (Corning Inc., Corning, NY, USA) containing 10% fetal bovine serum (FBS, Corning) and penicillin (100 U/mL, RMBIO, Missoula, MT, USA)/streptomycin (100 µg/mL, RMBIO) at 37°C with 5% CO_2_ in a humidified incubator. hsa-miR-340-5p mimics and negative control (NC mimics) were constructed by GenePharma (Shanghai, China) and transfected into ESCs to upregulate miR-340-5p. To overexpress MAP3K2, ESCs were transfected with pcDNA3.1/MAP3K2 or control pcDNA3.1 (GenePharma). Lipofectamine 2000 (Invitrogen, Carlsbad, CA, USA) was utilized for oligonucleotide or plasmid transfection. After 48 h, the transfection efficiency was evaluated by RT-qPCR.

### 2.2 Reverse transcription quantitative polymerase chain reaction (RT-qPCR)

Total RNA was isolated using TRIzol reagent (Invitrogen) from ESCs and was reverse transcribed into cDNA using a Bestar qPCR Reverse Transcription Kit (DBI^®^ Bioscience, Shanghai, China). RT-qPCR was implemented using SYBR Green qPCR Master Mix (DBI^®^ Bioscience) on an ABI7300 real-time PCR system (Applied Biosystems, Foster City, CA, USA). The quantification of miRNA and mRNAs was achieved with the 2^−ΔΔCt^ method, normalized to U6 and GAPDH, respectively. Primer sequences are provided in Table 1.

**Table 1:** Primer sequences used in RT-qPCR

Gene	Sequence (5′ → 3′)
hsa-miR-340-5p forward	CACTCCAGCTGGGTTATAAAGCAATGAGA
hsa-miR-340-5p reverse	TGGTGTCGTGGAGTCG
CYLD forward	CTCTTTACCATTCAGTCTCACC
CYLD reverse	CTCATCTTCCAGTTCCAGTCC
DMD forward	ACAGCTGGCATGGAAGATGAA
DMD reverse	ACGAGTTGATTGTCGGACCC
RPS6KA5 forward	TTGTGCTTGCCCTCGAACAT
RPS6KA5 reverse	CTGTAGGCAGACAAAACTTGCT
NFAT5 forward	TACCTCAGTCACCGACAGCAAG
NFAT5 reverse	CGACTGTTATCCAGCAAGTC
JPH1 forward	AATTAGGAAAGCCCCATCCG
JPH1 reverse	AAGGCAGCTGTTGACTTCCA
IPMK forward	GCACATGTACGGGAAGGACA
IPMK reverse	GGACAAGCTTTTGCCCACTG
SYDE2 forward	ACAGCCAATTCCCATGTCCA
SYDE2 reverse	TGTTGCAGTGTACCAGGACC
ESYT2 forward	CCGGGATCAGCGCGAG
ESYT2 reverse	GTGCTAAGGTGGGTGTTTGC
MAP3K2 forward	GCTTACGGTCTCCTGTGAGTT
MAP3K2 reverse	AGGATTGTCTATGTCACTTCCCC
GAPDH forward	GAGTCAACGGATTTGGTCGT
GAPDH reverse	TTGATTTTGGAGGGATCTCG
U6 forward	CTCGCTTCGGCAGCACA
U6 reverse	AACGCTTCACGAATTTGCGT

### 2.3 Western blotting

Total proteins were isolated from ESCs by RIPA buffer (Beyotime, Shanghai, China) and quantified with a BCA assay kit (Thermo Fisher Scientific, Waltham, MA, USA). Proteins (20 µg) were separated by 10% SDS-PAGE gels and transferred to polyvinylidene difluoride (PVDF) membranes (Roche, Mannheim, Germany). The membranes were blocked with 5% defatted milk and incubated with the primary antibodies as follows: anti-E-cadherin (ab40772, 1:10,000), anti-N-cadherin (ab76011, 1:5,000), anti-GAPDH (ab9485, 1:2,500), anti-MAP3K2 (ab33918, 1:10,000), anti-p-Erk1/2 (ab223500, 1:400), anti-Erk1/2 (ab184699, 1:10,000), anti-p-JNK (ab124956, 1:5,000), anti-JNK (ab199380, 1:2,500), anti-p-p38 (ab178867, 1:1,000), and anti-p38 (ab170099, 1:5,000) (all from Abcam, Cambridge, MA, USA) at 4°C overnight, followed by incubation with the horseradish peroxidase-conjugated secondary antibody of goat anti-rabbit IgG H&L (Abcam, ab175781, 1:10,000) at room temperature for 2 h. The proteins were visualized using an ECL kit (Cwbiotech, Beijing, China) and quantified with the Amersham Imager 600 (GE Healthcare Life Sciences, Little Chalfont, UK).

### 2.4 Transwell assay

A Transwell chamber (Corning) was used for assessing ESC migration and invasion. After 48 h of incubation, ESCs were washed and incubated with serum-free DMEM for 12 h. Afterward, the suspension (100 µL, 5 × 10^4^ cells) was added into the upper chambers and DMEM (500 µL) containing 10% FBS was placed into the lower chambers. After treatment for 24 h, a cotton swab was utilized to gently remove the nonmigratory cells. The migratory cells were fixed in 4% formaldehyde, stained with 0.1% crystal violet solution and counted under an Eclipse Ti-s microscope (Olympus, Tokyo, Japan). Invasion assay was conducted similar to the above migration assay, except that Matrigel (Corning) was precoated for the chambers.

### 2.5 Luciferase reporter assay

The putative binding site between miR-340-5p and MAP3K2 was predicted by TargetScan (http://www.targetscan.org/vert_71/). Wild type (Wt) or mutant (Mut) 3′untranslated region (3′UTR) of MAP3K2 was inserted into pmirGLO vectors (Promega, Madison, WI, USA). These vectors were co-transfected with miR-340-5p mimics or NC mimics into ESCs using Lipofectamine 2000 (Invitrogen). Measurement of the luciferase activity was performed with a dual luciferase^®^ reporter assay system (Promega).

### 2.6 Statistical analysis

SPSS 20.0 software (IBM Corp, Armonk, NY, USA) was used for data analysis. Specific data are provided in supplementary Table 2. All numerical results are expressed as the mean ± standard deviation. Comparisons between two groups were evaluated by Student’s *t*-test, and those among more groups were assessed by analysis of variance (ANOVA) followed by Tukey’s *post hoc* test. Each experiment was performed at least three times. *p* < 0.05 was considered statistically significant.

**Table 2:** Data from SPSS analysis

	Value 1	Value 2	Value 3	Average	SD	*p* value
**Figure 1a**						
NC mimics	0.91	0.96	1.12	1	0.11	1.28 × 10^−04^
miR-340-5p mimics	3.87	4.26	4.61	4.25	0.37	
**Figure 1c**						
Migration						
NC mimics	162.00	178.00	191.00	177	14.45	3.47 × 10^−04^
miR-340-5p mimics	59.00	70.00	75.00	68	7.98	
Invasion						
NC mimics	128.00	140.00	158.00	142	15.39	2.73 × 10^−04^
miR-340-5p mimics	31.00	36.00	37.00	35	3.22	
**Figure 1d**						
E-cadherin						
NC mimics	0.89	0.97	1.14	1	0.13	0.006
miR-340-5p mimics	1.59	1.75	2.03	1.79	0.22	
N-cadherin						
NC mimics	0.90	0.98	1.12	1	0.11	3.24 × 10^−04^
miR-340-5p mimics	0.23	0.24	0.27	0.25	0.021	
**Figure 2b**						
CYLD						
NC mimics	0.92	0.96	1.11	1	0.1	0.733
miR-340-5p mimics	0.92	1.04	1.12	1.03	0.1	
DMD						
NC mimics	0.90	0.98	1.12	1	0.11	0.819
miR-340-5p mimics	0.92	0.94	1.08	0.98	0.09	
RPS6KA5						
NC mimics	0.88	0.98	1.14	1	0.13	0.691
miR-340-5p mimics	0.87	0.95	1.06	0.96	0.098	
NFAT5						
NC mimics	0.91	1.01	1.09	1	0.09	0.946
miR-340-5p mimics	0.91	0.96	1.16	1.01	0.13	
JPH1						
NC mimics	0.95	1.00	1.05	1	0.05	1
miR-340-5p mimics	0.91	0.97	1.12	1	0.11	
IPMK						
NC mimics	0.93	0.96	1.11	1	0.1	0.903
miR-340-5p mimics	0.91	0.97	1.09	0.99	0.09	
SYDE2						
NC mimics	0.92	0.99	1.08	1	0.08	0.606
miR-340-5p mimics	0.92	1.05	1.16	1.04	0.12	
ESYT2						
NC mimics	0.94	0.99	1.08	1	0.07	0.824
miR-340-5p mimics	0.94	0.99	1.13	1.02	0.1	
MAP3K2						
NC mimics	0.89	0.97	1.14	1	0.13	0.002
miR-340-5p mimics	0.41	0.43	0.49	0.44	0.04	
**Figure 2d**						
NC mimics	0.92	1.00	1.08	1	0.08	0.001
miR-340-5p mimics	0.48	0.54	0.58	0.53	0.05	
**Figure 2f**						
Wt						
NC mimics	0.92	0.98	1.10	1	0.09	3.70 × 10^−04^
miR-340-5p mimics	0.35	0.38	0.41	0.38	0.032	
Mut						
NC mimics	0.92	0.96	1.12	1	0.11	0.713
miR-340-5p mimics	0.86	0.98	1.06	0.97	0.1	
**Figure 3b**						
Empty	0.93	0.96	1.11	1	0.1	0.001
MAP3K2	2.52	2.94	3.12	2.86	0.31	
**Figure 3d**						
Migration						
NC mimics	154.00	158.00	183.00	165	15.47	
miR-340-5p mimics	53.00	62.00	65.00	60	5.93	5.79 × 10^−05^
miR-340-5p mimics + MAP3K2	105.00	89.00	91.00	95	8.8	0.019
Invasion						
NC mimics	114.00	118.00	133.00	122	10.02	
miR-340-5p mimics	31.00	34.00	37.00	34	3.44	1.50 × 10^−05^
miR-340-5p mimics + MAP3K2	71.00	82.00	84.00	79	7.45	0.001
**Figure 3e**						
E-cadherin						
NC mimics	0.88	0.98	1.14	1	0.13	
miR-340-5p mimics	2.93	3.11	3.64	3.23	0.37	9.55 × 10^−05^
miR-340-5p mimics + MAP3K2	1.69	1.86	2.09	1.88	0.2	0.002
N-cadherin						
NC mimics	0.91	0.99	1.11	1	0.1	
miR-340-5p mimics	0.17	0.20	0.20	0.19	0.02	1.38 × 10^−05^
miR-340-5p mimics + MAP3K2	0.43	0.45	0.53	0.47	0.053	0.005
**Figure 4b**						
p-Erk1/2/Erk1/2						
NC mimics	0.93	0.98	1.09	1	0.08	
miR-340-5p mimics	0.29	0.31	0.35	0.32	0.031	3.04 × 10^−05^
miR-340-5p mimics + MAP3K2	0.52	0.55	0.65	0.57	0.07	0.006
p-JNK/JNK						
NC mimics	0.92	0.95	1.13	1	0.11	
miR-340-5p mimics	0.42	0.49	0.49	0.47	0.04	0.001
miR-340-5p mimics + MAP3K2	0.87	0.92	1.03	0.94	0.084	0.001
p-p38/p38						
NC mimics	0.89	0.98	1.13	1	0.12	
miR-340-5p mimics	0.37	0.39	0.44	0.4	0.037	1.89 × 10^−04^
miR-340-5p mimics + MAP3K2	0.60	0.61	0.68	0.63	0.045	0.025


**Ethical approval:** Our study did not require an ethical board approval because it did not contain human or animal trials.

## 3 Results

### 3.1 miR-340-5p inhibits cell migration, invasiveness and EMT in ESCs

**Figure 1:**
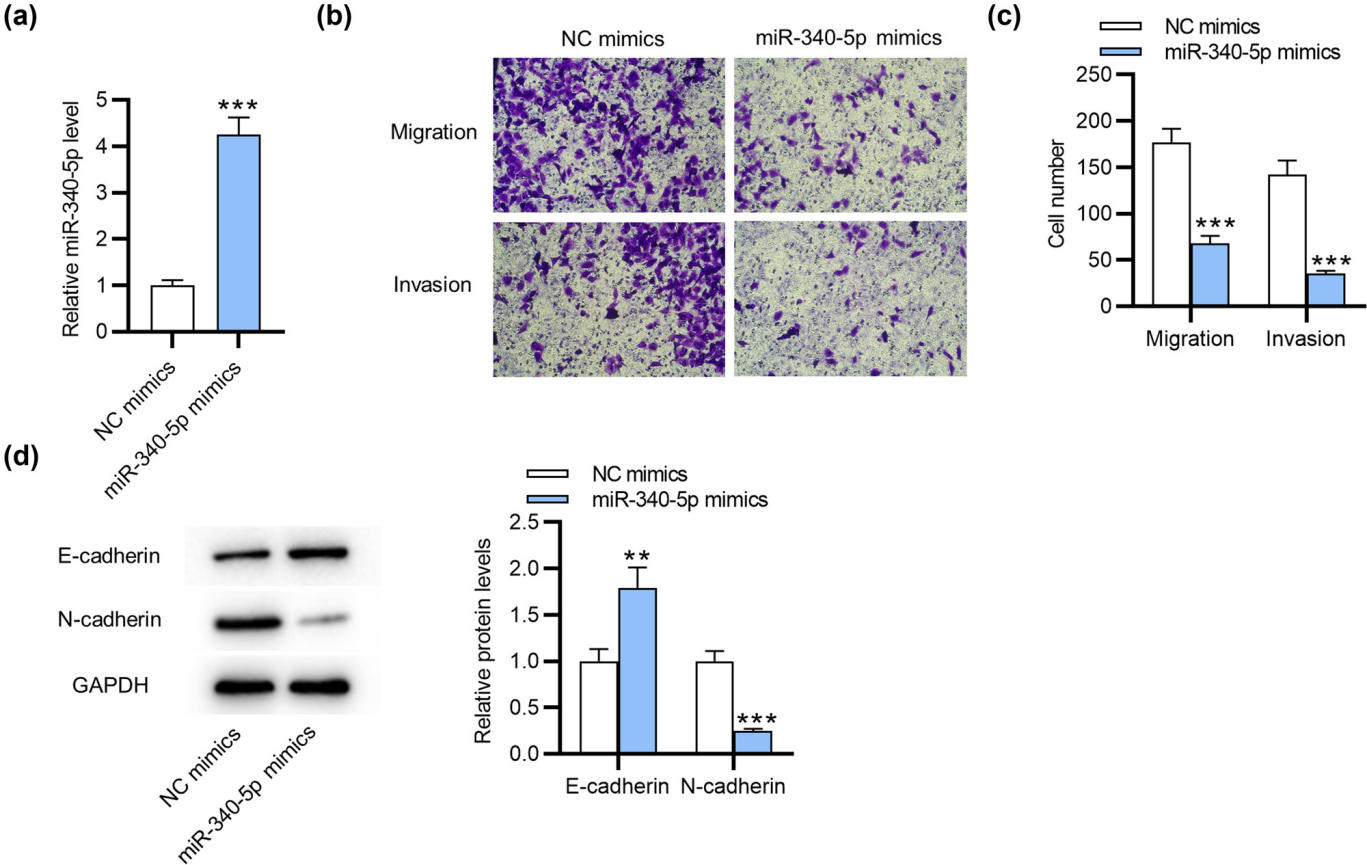
miR-340-5p restrains cell migration, invasiveness and EMT in endometriosis. (a) RT-qPCR for the transfection efficiency of miR-340-5p mimics in ESCs. (b and c) Transwell assays for evaluating ESC migratory and invasive abilities after overexpressing miR-340-5p. (d) Western blotting for assessing protein levels of EMT-associated markers (E-cadherin and N-cadherin). **p < 0.01, ***p < 0.001.

To determine the role of miR-340-5p in endometriosis, we first overexpressed miR-340-5p. As shown by RT-qPCR, the miR-340-5p level was significantly enhanced after transfection with miR-340-5p mimics (Figure 1a). Afterward, we performed Transwell assays, which displayed that overexpressing miR-340-5p restrained the migratory ability of ESCs as well as the invasive ability (Figure 1b and c). Moreover, western blotting suggested that miR-340-5p upregulation increased the protein level of E-cadherin but reduced that of N-cadherin (Figure 1d). This suggested that miR-340-5p restrains EMT process in ESCs.

### 3.2 miR-340-5p directly targets MAP3K2

**Figure 2:**
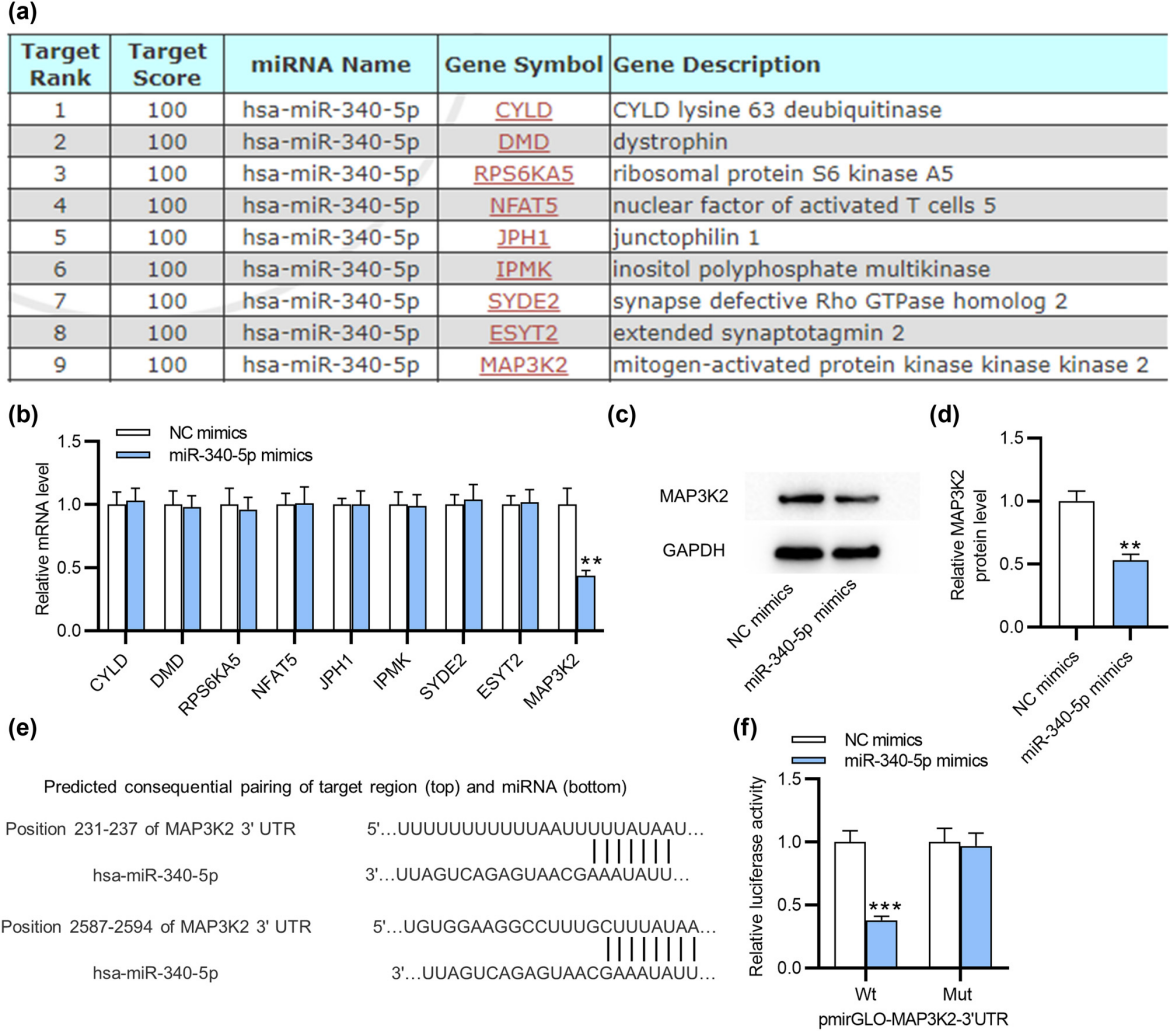
miR-340-5p targets MAP3K2. (a) MiRDB was used for screening the downstream targets of miR-340-5p. (b) RT-qPCR of the potential mRNA levels in ESCs transfected with miR-340-5p or NC mimics. (c and d) Western blotting of MAP3K2 protein expression in ESCs with above transfection. (e) Bioinformatics analysis of the potential complementary site of miR-340-5p on MAP3K2. (f) The luciferase reporter assay revealed luciferase activity of Wt/Mut pmirGLO-MAP3K2-3′UTR in ESCs after upregulating miR-340-5p. **p < 0.01, ***p < 0.001.

To clarify how miR-340-5p exerts its impact on endometriosis progression, we used miRDB (http://mirdb.org/mirdb/index.html) for identifying the downstream targets of miR-340-5p and selected the top nine mRNAs with 100 binding scores (Figure 2a). Subsequently, we implemented RT-qPCR to detect the levels of these mRNAs in ESCs and the results indicated that only MAP3K2 level was markedly decreased by miR-340-5p mimics (Figure 2b). Additionally, the MAP3K2 protein level was reduced by miR-340-5p mimics, as revealed by western blotting (Figure 2c and d). Bioinformatics analysis with TargetScan elucidated the complementary site of miR-340-5p on MAP3K2 3′UTR (Figure 2e). To substantiate the relationship between MAP3K2 and miR-340-5p, the luciferase reporter assay was conducted. miR-340-5p mimics weakened the luciferase activity in the MAP3K2-Wt group, whereas it almost had no impact on the MAP3K2-Mut group (Figure 2f). Collectively, MAP3K2 is targeted by miR-340-5p in ESCs.

### 3.3 Upregulation of MAP3K2 abolishes miR-340-5p upregulation-mediated suppressive impact on the invasive behaviors of ESCs

**Figure 3:**
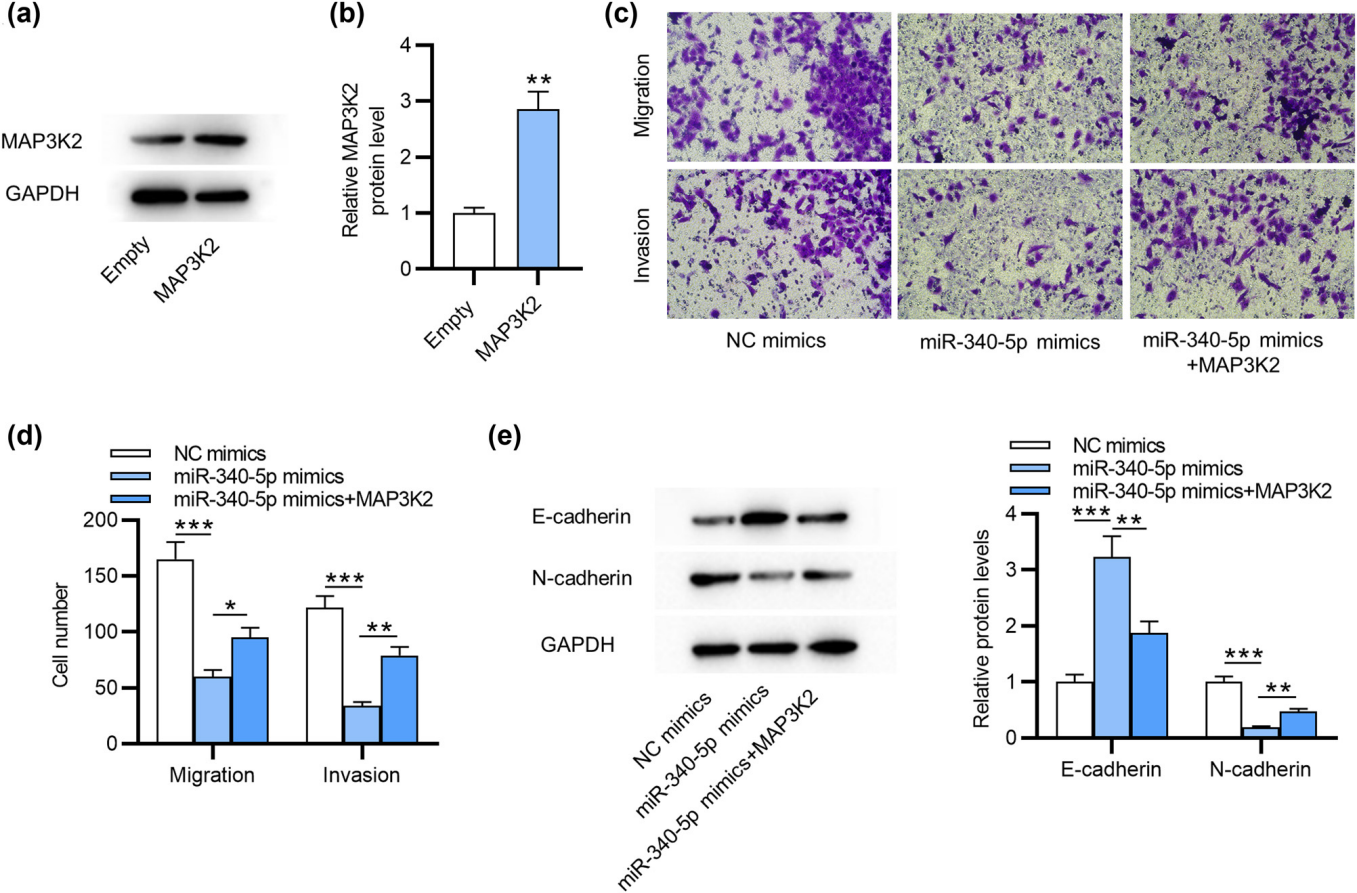
MAP3K2 overexpression abolishes miR-340-5p overexpression-mediated suppressive impact on the invasive behaviors of ESCs. (a and b) Western blotting of MAP3K2 protein level in ESCs after overexpressing MAP3K2. (c and d) Measurement of the migration and invasion by Transwell assays in ESCs transfected with NC mimics, miR-340-5p mimics or miR-340-5p mimics + pcDNA3.1/MAP3K2. (e) Western blotting of E-cadherin and N-cadherin protein levels in ESCs with the above transfection. *p < 0.05, **p < 0.01, ***p < 0.001.

Subsequently, we explored the detailed effects of MAP3K2 on miR-340-5p in ESCs. As displayed by western blotting, MAP3K2 protein expression was significantly increased after transfection of pcDNA3.1/MAP3K2 in ESCs (Figure 3a and b). Furthermore, Transwell assays demonstrated that overexpressing MAP3K2 abolished the suppressive influence on cell migration and invasiveness caused by miR-340-5p mimics (Figure 3c and d). The levels of EMT-associated markers were examined by western blotting, which suggested that E-cadherin expression enhanced by miR-340-5p mimics was downregulated after upregulating MAP3K2 (Figure 3e). Similarly, MAP3K2 upregulation reversed the level of N-cadherin that was reduced by miR-340-5p upregulation (Figure 3e). In summary, MAP3K2 restoration rescues the miR-340-5p overexpression-induced suppressive impact on the invasive characters of ESCs.

### 3.4 miR-340-5p regulates the MAPK/ERK signaling pathway by targeting MAP3K2

**Figure 4:**
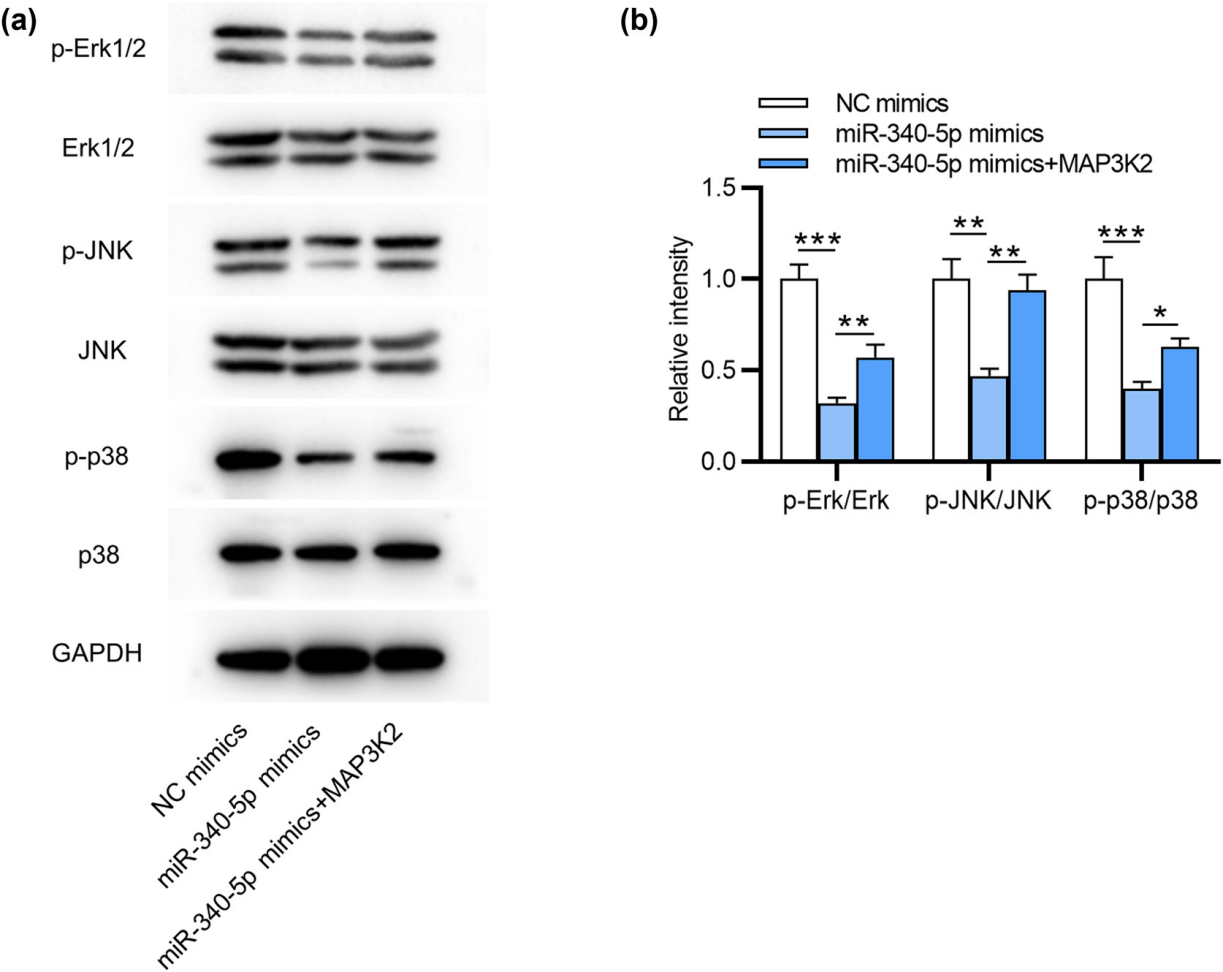
MiR-340-5p represses endometriosis progression by regulating MAP3K2-mediated MAPK/ERK pathway. (a and b) Western blotting of MAPK/ERK signaling pathway-related protein levels in ESCs. *p < 0.05, **p < 0.01, ***p < 0.001.

MAP3K2 is able to activate other kinases involved in the MAPK/ERK signaling pathway [23]. Here, we detected MAPK/ERK signaling pathway-associated proteins in ESCs. As displayed by western blotting, overexpressing miR-340-5p reduced the ratios of p-Erk to total Erk, p-JNK to total JNK and p-p38 to p38, and this effect was then partially reversed by upregulating MAP3K2 (Figure 4a and b). Hence, miR-340-5p influences the progression of endometriosis by regulating MAP3K2-mediated MAPK/ERK signaling.

## 4 Discussion

Emerging evidence has indicated that endometriosis is a precancerous lesion, which exhibits cancer-like characterizations such as cell invasiveness and uncontrolled cell proliferation [26]. Patients with endometriosis, particularly ovarian endometriosis, have an increased risk of developing ovarian cancer [27]. Many factors are considered to be implicated in the pathogenesis of endometriosis, including environmental and genetic factors, immune response and hormonal effects [28,29]. Infiltration of immune cells and excessive secretion of proinflammatory cytokines are observed in the peritoneal cavity of women affected by endometriosis [30,31]. Moreover, previous studies have demonstrated that upregulation of genes related to cell adhesion and extracellular matrix precedes the formation of endometriotic lesions [32]. All the above factors are shown to contribute to the invasive behaviors of endometriotic cells including proliferation, invasion, adhesion and survival, which consequently leads to endometriosis [33].

MiRNAs have been increasingly indicated to be a crucial regulator in the development of diverse human diseases [34,35,36,37]. Numerous studies have confirmed that miR-340-5p exerts an indispensable impact on multiple diseases. For example, miR-340-5p upregulation improves spinal cord injury-induced apoptosis and neuroinflammation via regulating the p38/MAPK pathway [38]. miR-340-5p targets PDCD4 to protect from a brain injury after intracerebral hemorrhage [39]. Furthermore, a previous study has elucidated the decreased expression of miR-340-5p in patients with endometriosis [40]. EMT is considered as a key factor in a variety of pathological processes, including tumor metastasis and invasiveness [41]. Numerous studies have verified that EMT is a prerequisite for endometriosis since ectopic lesions in endometriosis display similar biological properties as cancer metastasis [13]. In the process of EMT, cells gain increased migratory and invasive properties, consequently contributing to the development of endometriotic cells in ectopic sites [42]. In the present study, we examined the specific function of miR-340-5p in ESCs. As revealed by the results, overexpressed-miR-340-5p had a suppressive impact on the migration, invasiveness and EMT of ESCs. This indicates that miR-340-5p might protect against the formation of endometriosis, which is in accord with the previous study.

MiRNAs are well-known to exert their regulatory impacts on gene expression by targeting mRNA 3′UTRs, subsequently causing either translational repression or mRNA degradation [43]. With the assistance of bioinformatics tools, MAP3K2 was identified as the target of miR-340-5p. MAP3K2 is implicated in diverse cellular processes, including cell differentiation, migration and proliferation [44]. Notably, in this study, overexpressing MAP3K2 attenuated the suppressive impact on the invasive characters of ESCs caused by miR-340-5p upregulation. Furthermore, MAP3K2 has been verified to participate in various pathways, including the MAPK/ERK signaling pathway [45]. MAP3K2 is able to activate several downstream kinases of the MAPK signaling pathway, including Erk1/2, JNK and p38 [46]. In this study, we analyzed the levels of these kinases in ESCs with indicated treatment. As anticipated, the phosphorylation of Erk, JNK and p38 was markedly reduced by miR-340-5p mimics and this effect was then rescued by overexpressing MAP3K2, revealing that miR-340-5p inactivates MAPK/ERK signaling by targeting MAP3K2 in ESCs.

## 5 Conclusion

In conclusion, we investigated the function and mechanism of miR-340-5p in ESCs. As indicated by the results, miR-340-5p inhibits the migration, invasiveness and EMT in ESCs, which can be reversed by its downstream target MAP3K2. miR-340-5p inactivates the MAP3K2-mediated MAPK/ERK signaling in ESCs. Our findings might provide a new perspective for the treatment of patients with endometriosis. However, there are still limitations in this study. To better understand the role of miR-340-5p in endometriosis, *in vivo* experiments are demanded in future studies. Additionally, further investigations are needed to have a better understanding of the pathogenesis of endometriosis.
